# EHMTI-0323. Pediatric aspects of venous headache. The indicators of cerebral venous outflow for diagnostics and treatment of children

**DOI:** 10.1186/1129-2377-15-S1-B1

**Published:** 2014-09-18

**Authors:** M Abramova, S Novoselova, I Stepanova, N Shurupova

**Affiliations:** 1 Neurology neurosurgery and medical genetics Department, Pirogov Russian National Research Medical University, Moscow, Russia

## 

Disturbances of cerebral venous hemodynamics define development of child headache. However directed by the diagnosis and subsequent treatment these disturbances are not considered. The absence of diagnostics algorithm and norms of cerebral venous blood flow limits the possibility of timely therapy at children.

## Aims

The studies of headache caused by venous hemodynamics disturbance.

## Materials and methods

600 patients (3-17 years old) who complained of headache have been examined. The data of a blood flow velocity in deep cerebral veins of a brain: in straight venous sinus, vein of Galen, sinuses cavernous defined by Transcranial Color-Coded Duplex.

## Results

Headache caused by cerebral venous dysfunction notes in 47% of children. The surveyed children had typical headache (100%) of holding apart character in occipital and parietal areas after a dream (69%) ,after physical activity(14%) and a long static pose have been revealed(17%).“Venous” headache at children often can be similar to dizziness. The attacks of headache which are coming to an end with vomiting are revealed at 25% of children. Children also complained of nasal bleeding as a fountain (60%) during a night or day dream (40%), noise in ears (53%), ocular pathology refraction(43%) and expressed vegetative symptoms (80%).

We have found clinical signs of the connective tissue dysplasia syndromes at 67% of children.

Structural cerebral abnormalities (hypoplasia of cerebral venous sinuses) were revealed at 9% patients of them by MRI .

“Markers” of disturbances in cerebral venous hemodynamics was venous outflow in deep brain veins. The research of parameters of a cerebral hemodynamics was carried out in the conditions of functional rest and after dynamic tests. We suggest modified Valsalva, orthostasis and head-down tilting tests in children .

## Conclusion

Definition of cerebral venous hemodynamic normal indicators in children of different age groups is very important for identification and treatment of headache with cerebral venous disturbances.

No conflict of interest.

